# VvBAP1, a Grape C2 Domain Protein, Plays a Positive Regulatory Role Under Heat Stress

**DOI:** 10.3389/fpls.2020.544374

**Published:** 2020-11-09

**Authors:** Qing Ye, Jintao Yu, Zhen Zhang, Lixia Hou, Xin Liu

**Affiliations:** Key Lab of Plant Biotechnology in University of Shandong Province, College of Life Science, Qingdao Agricultural University, Qingdao, China

**Keywords:** *Vitis vinifera* L., heat stress, *VvBAP1*, reactive oxygen species, antioxidant enzyme

## Abstract

Temperature is considered one of the critical factors directly influencing grapevine during the three primary growth and development stages: sprout, flowering, and fruit-coloring, which is strongly correlated to the yield and quality of the grape. The grapevine is frequently exposed to high-temperature conditions that are detrimental to growth. However, the mechanisms of the heat stress response and adaptation in grapevine are not adequately studied. The *Arabidopsis* copine gene *AtBON1* encodes a highly conserved protein containing two C2 domains at the amino terminus, participation in cell death regulation and defense responses. Previously, we showed that a BON1 association protein from the grapevine, *VvBAP1*, plays a positive role in cold tolerance. Similarly, the involvement of *VvBAP1* in the resistance to heat stress was also found in the present study. The results indicated *VvBAP1* was significantly induced by high temperature, and the elevated expression of *VvBAP1* was significantly higher in the resistant cultivars than the sensitive cultivars under heat stress. Seed germination and phenotypic analysis results indicated that overexpression of *VvBAP1* improved *Arabidopsis* thermoresistance. Compared with the wild type, the chlorophyll content and net photosynthetic rate in *VvBAP1* overexpressing *Arabidopsis* plants were markedly increased under heat stress. At high temperatures, overexpression of *VvBAP1* also enhanced antioxidant enzyme activity as well as their corresponding gene transcription levels, to reduce the accumulation of reactive oxygen species and lipid peroxidation. Besides, the transcriptional activities of *HSP70*, *HSP101*, *HSFA2*, and *HSFB1* in *VvBAP1* overexpressing *Arabidopsis* plants were significantly up-regulated compare to the wild type. In summary, we propose that *VvBAP1* may play a potential important role in enhanced grapevine thermoresistance, primarily through the enhancement of antioxidant enzyme activity and promoted heat stress response genes expression.

## Introduction

Temperature is considered one of the critical factors directly influence grapevine more the three primary growth and development stages: sprout, flowering and fruit-coloring (Lorenz et al., 1995). Both yield and quality of grapevine are reduced when encountered high temperatures (Wahid et al., 2007). Therefore, high temperature is one of the principal limiting factors in the development of grapevine economic industry worldwide. Through climate prediction models, the Intergovernmental Panel on Climate Change predicted an increase in global mean temperatures between 1.5°C and 5°C during the 21st century. Also, global warming will likely accompany with more frequent and powerful extreme temperature events (Lobell et al., 2008). Thus, revealing the mechanism of the grapevine thermoresistance has become a vital research topic that provides a reliable theoretical basis for grapevine breeding.

When exposed to a high-temperature conditions, a series of injuries in plants, including protein misfolding and denaturation, irreversible loss of enzyme activity, and disruption of cellular structural components, occur (Schöffl et al., 1998; Howarth, 2005). These damaging events ultimately caused a severe reduction in the net photosynthetic rate and ion flux, excessive production of reactive oxygen species (ROS), such as hydrogen peroxide (H_2_O_2_) and superoxide $\left({\text{O}}_{2}^{-}\right)$, thus inhibition of plant growth (Bokszczanin et al., 2013). To maintain metabolic homeostasis under heat stress that the organisms can survive and even multiply, plants have formed physiological, biochemical, cellular, and molecular regulatory mechanisms to precisely regulate thermoresistance (Bartels and Sunkar, 2005; Zhuang et al., 2014).

As ROS induced by heat stress may cause oxidative damage, plants have also evolved a unique ROS scavenging systems, like superoxide dismutase (SOD), catalase (CAT), ascorbate peroxidase (APX), peroxidase (POD) and other antioxidant enzymes, might work with synergy to reduce accumulation of cellular ROS and attenuate oxidative injury to plants (Mittler et al., 2011). Additionally, more and more results have indicated that heat shock proteins (HSPs) are important molecular chaperones that ensure protein proper folding, which are essential to help plants acquire thermotolerance (Chen et al., 2011). Moreover, the small HSP (sHSP) and heat stress transcription factors (HSFs) were also reported to help plant growth and development under heat stress (Qu et al., 2013).

Grape (*Vitis vinifera* L.) is one of the most essential economic fruit crops throughout the world. In recent years, China has the most productive and second most widely cultivated area of grapevine worldwide. However, the fruit yield and quality of grapevine are often highly influenced by extreme climatic conditions, especially heat stress (Szenteleki et al., 2012). In many main centers of origin for grapevine, the daily maximum temperature can often surpass 40°C, even beyond 45°C, which has seriously limited the development of the grapevine economic industry (Salazar-Parra et al., 2010; Pillet et al., 2012). However, in elucidating the response and adaptation of grapevine to heat stress, immense efforts have been put into exploring the physiological and morphological changes. Recently, many studies have offered unique insights into understanding the heat stress responses of grapevine *via* transcriptomic and proteomic analyses (Kosová et al., 2011; Jiang et al., 2017). In grapevine leaves, several sHSPs and APX encoding genes identified as playing significant roles in thermotolerance (Liu et al., 2012). Previous transcriptome data have revealed high transcript levels of a series of *VvHSF* genes such as *VvHSFA1a*, *VaHSFA1a*, *VvHSFA2a*, *VaHSFA2a*, and *VvHSFB2b* in *Vitis vinifera* or *Vitis amurensis*, permitting adaptation to heat stress (Liu et al., 2012; Xin et al., 2013; Rocheta et al., 2014; Xu et al., 2014). Additionally, *VvHSFA2a* expression is up-regulated in grapevine berries during heat stress (Pillet et al., 2012). Similarly, it has been demonstrated that the *VvHSFB2b* homologous gene *VpHSFB2b* is related to heat resistance in Chinese wild grape *Vitis pseudoreticulata* (Peng et al., 2013). Hu et al. (2016) reported ten *VpHSFs*, especially *VpHSFB1a*, *VpHSFC1a*, *VpHSFA2a*, *VpHSFA3a*, and *VpHSFA6a* were markedly up-regulate exposed to heat stress, the results suggest that their positive regulation roles of heat stress responsive in *Vitis pseudoreticulta*. Furthermore, the transcription of galactinol synthase gene, *VvGOLS1* was detected significantly as up-regulated in grape berries under high temperature conditions (Pillet et al., 2012). Although numerous genes that relate to grapevine thermotolerance have been recognized, knowledge about the precise functions and molecular mechanism are largely unknown at the present time.

In *Arabidopsis*, AtBAP1 (BON1 ASSOCIATED PROTEIN 1) has been demonstrated to belong to C2 domain phospholipid-binding protein as a functional partner of AtBON1 (BONZAI1). AtBON1 encodes a highly conserved protein containing two C2 domains at the N-terminus, is involved in cell death regulation and defense responses (Hua et al., 2001). *Arabidopsis AtBON1*-deficient mutant displayed reduced plant height at the optimum growth temperature, and that overexpression of *AtBAP1* could compensate for the dwarf phenotype of the *bon1-1* mutant, indicating these two proteins have similar biological functions (Hua et al., 2001; Yang and Hua, 2004). Subsequent study has shown that *AtBAP1* could suppress programmed cell death induced by virulent pathogens and ROS (Yang et al., 2007). Furthermore, the transcription factor AtICE1 can bind to the promoter of *AtBAP1*, thus promoting the elevated transcription level of *AtBAP1* under cold stress (Zhu et al., 2011). However, the biological role of *BAP1* in grapevine has not been fully elucidated.

In our previous study, we cloned and functionally characterized *VvBAP1* from the grapevine ‘F-242’ as the nearest orthologue to *AtBAP1* (Zhang et al., 2014). Then, we found that *VvBAP1* was correlated with the cold resistance in grapevine, *VvBAP1* could regulate the soluble sugar content and enhance antioxidant enzyme activities, thereby promoting the grape cold resistance (Hou et al., 2018). Recently, Cao et al. (2019) demonstrated that *VvBAP1* may functioning as an important factor in suppressing grape berries cell death, its transcript was significantly inhibited by drought stress. However, further studies should be conducted to examine whether *VvBAP1* involved in the heat tolerance of grapes. Thus, we evaluated *VvBAP1* expression in cultivars of grapevine that are known to exhibit different responses to heat stress conditions. Further, the *VvBAP1*-overexpressing *Arabidopsis* plants were used to analyze the physiological functions of *VvBAP1* by measuring a series of physiological indexes relevant to heat stress response, with an aim of revealing the *VvBAP1*-mediated mechanisms which are inducing thermoresistance in grapes.

## Materials and Methods

### Plant Materials and Growth Conditions

Shoots (with buds) of four grape (*Vitis vinifera* L.) cultivars named ‘Chardonnay’, ‘Cabernet Sauvignon’, ‘Zuoyouhong’ and ‘Beta’ served as explants. The shoots were rinsed with water overnight, and surface sterilized with 75% (v/v) ethanol for 30 s, followed by 0.1% HgCl_2_ for 8 min. After that, the explants were washed 3-5 times with sterile water. Next, a pair of scissors was used to cut approximately 2-3 cm of stem segment, leaving the apical bud intact. The explants were cultured on sterile MS solid medium (half strength) containing 0.57 μM IAA (indole acetic acid). Culture conditions were as follows: 12 h light/12 h dark cycle (light intensity of 200 μmol·m^-2^·s^-1^); 25 ± 1°C. The tissue culture seedlings were used in experiments after 40-55 days.

Wild type *Arabidopsis thaliana* used herein was of the ecotype Columbia (Col-0). The transgenic *Arabidopsis* plants overexpressing *VvBAP1* (*OEVvBAP1-38* and *OEVvBAP1-40*) were described in our previous study (Hou et al., 2018). Regarding plant growth, we surface sterilized the *Arabidopsis* seeds and maintained them at 4°C for 72 h. After that, they were germinated and cultured on sterile MS solid medium at 22 ± 1°C under a 16 h light/8 h dark cycle (light intensity of 120 μmol·m^-2^·s^-1^).

### Heat Stress Treatment

To test the response of *VvBAP1* to high temperature, the 45-55 days old grape subcultured seedlings were placed in a growth chamber at 40°C for 0, 3, 6, 9, 12, 18, and 24 h. Control plants were maintained at 25 ± 1°C. At the end of each time point, the leaves were sampled then preserved in liquid nitrogen for RNA extraction.

Regarding *Arabidopsis* seed germination analysis, the seeds were maintained for 6 h at 45°C prior to germination. Seeds for each genotype were sown (300 per plate) on the same MS solid medium and maintained at 22°C under constant light (60 μmol·m^-2^·s^-1^). The seeds were not received, 45°C treatment was used as the control. Seeds that exhibited a clear protrusion of the radicle *via* the seed coat were considered germinated. The number of germinated seeds were recorded after every 12 h during the experiment.

Seven-day old seedlings of each genotype *Arabidopsis* were placed at 45°C for 2 h, then maintained them to continue to cultivate for 2-3 days at 22°C. The 4 weeks phase seedlings of each genotype *Arabidopsis* were treated at 42°C for 8 h, then plants were recovered to grow at 22°C for 10 days. Then the phenotypes were observed, and the survival rates were measured. Control plants were cultured at 22°C ± 2°C.

The 4 weeks phase seedlings of each genotype *Arabidopsis* were treated at 45°C for 2 h, then the change electrolyte leakage, malondialdehyde (MDA) content, SOD, POD, CAT and APX activities, expression levels of *Cu/Zn SOD, POD2, CAT1*, *CAT2, CAT3*, *APX1*, *APX2* and heat response-related gene were tested.

### qRT-PCR

We employed the CTAB method (Iandolino et al., 2004) to extract total RNA from the leaves of the experimental plants. The RNA was then reverse transcribed to cDNA using the Prime Script RT reagent Kit with the Gdna Eraser (TaKaRa, Dalian, China). The MyiQ Real-Time PCR Detection System (Bio-Rad, USA) was used to perform RT-PCR. SYBR green I (BioWhittaker Molecular Applications) was included in the reaction master mix. The following reaction conditions were used: 95°C for 60 s; 40 cycles of 95°C for 10 s; 56°C for 20 s; and 72°C for 15 s. Each experiment containing three replicates was repeated at least thrice. Relative gene expression was determined using the 2^-ΔΔCT^ method (Livak and Schmittgen, 2001). Genes *AtACTIN2* or *VvACTIN* served as internal control for *Arabidopsi* and grape, respectively. **Table S1** shows the primers used in the qRT-PCR experiments.

### MDA Content and Electrolyte Leakage

MDA content was estimated by the method as described previously (Ding et al., 2007). Briefly, 0.1 g leaves were ground into homogenate in 1 ml 10% (w/v) trichloroacetic acid (TCA), and then supernatant was collected by centrifugation for 10 min at 4000 rpm. Next, 500 μl of the supernatant was added to equal volume of 10% (w/v) TCA, containing 0.6% (w/v) thiobarbituric acid (TBA). The mixture was then incubated at 100°C for 15 min, and then centrifuged for 10 min at 4000 rpm after cooling to room temperature. The absorbance of the mixture was measured at 532 nm then adjusted at 600 nm for non-specific absorbance. The quantity of MDA was computed from the extinction coefficient of 155 mM^−1^ cm^−1^ and presented as μmol kg^−1^, in which one unit was equivalent to 1 μmol MDA per kg of pulp.

Electrolyte leakage from the leaf discs were determined as per the methods described previously (Zhao et al., 2009), with a few variations. In brief, we rinsed the treated leaves with deionized (DI) water and left then to dry. Next, the leaf discs were obtained using a circular borer, and then soaked in DI water at 25°C for 1 h. The electrical conductivity (EC1) of the leakage solution from the leaf discs was detected with a conductivity meter (YSI model 55). Then, the mixture was brought to a boil for 10 min. Both total ionic strength and the electrical conductivity (EC2) were measured after cooling the solution to room temperature. The formula below was used to calculate the relative permeability of the membrane: EC1/EC2 × 100%.

Each experiment contained three biological replicates and was repeated at least thrice.

### Measurement of Chlorophyll Content and Photosynthetic Rate

A portable chlorophyll meter (SPAD-502PLUS, Minolta, Tokyo, Japan) was used to measure the chlorophyll content. The rate of photosynthesis was assessed using a liquid-phase oxygen measurement system (CHLOPOLAB-2, Hansatech, King’s Lynn, UK), following the instructions provided by the manufacturer. While taking the measurements, plants were maintained in 200 μmol m^−2^ s^−1^ light intensity at 25°C.

### Detection of Reactive Oxygen Species (ROS)

Reactive oxygen species (ROS) accumulation was determined by assessing the levels of superoxide $\left({\text{O}}_{2}^{-}\right)$ and hydrogen peroxide (H_2_O_2_) *via* histochemical staining. We performed 3,3’ -diaminobenzidine (DAB) and nitro-blue tetrazolium (NBT) staining according to previous publication (Wang K. et al., 2017). Each treatment, rosette leaves were picked and soaked in DAB (1 mg mL^–1^, pH 3.8) or NBT solution (0.1 mg mL^-1^) at 25°C for 8 h, and then photographed and subjected to analysis after sufficient bleaching in boiling 75% (v/v) ethanol. The quantitative measurements of H_2_O_2_, $\left({\text{O}}_{2}^{-}\right)$ were performed according to Zhang et al. (2011). At least three experiments were performed, each experiment contained three biological replicates.

### Measurement of Antioxidant Enzyme Activity

The frozen leaves samples (0.6 g) were ground into homogenate in ice-cooled 0.1 M phosphate buffer (pH 7.6) containing 0.5 mM EDTA, and then supernatant was obtained by centrifugation at 12,000 rpm for 10 min at 4°C. The supernatant was collected to determinate antioxidant enzymes activities. CAT activity was measured by the previously reported protocol (Aebi, 1984), and absorbance was taken at 240 nm. The activity of SOD and POD were investigated according to the Liang et al. (2015) method with minor changes. The activity of APX was evaluated as per the methods described previously (Wang L. et al., 2017). At least three experiments were performed, each experiment was performed in biological triplicate. The result from one set of experiments is provided here.

### Statistical Analysis

Statistical analysis for all experiments were performed using SAS. Differences between multiple treatments were analyzed using one-way ANOVA and means separated by Tukey’s HSD test (P < 0.05). Data are mean values of three independent biological replicates ± SE.

## Results

### Expression Profiles of *VvBAP1* in Grape Cultivars Leaves With Different Thermotolerance

**Figure 1:**
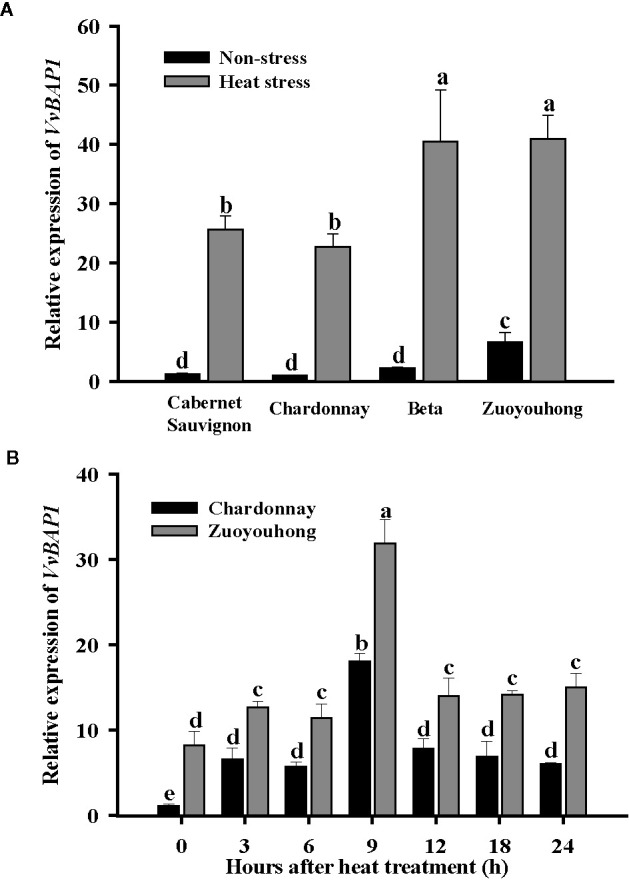
Expression profiles of *VvBAP1*. **(A)** Relative transcript level of *VvBAP1* in leaves of different grape varieties with 40°C treatment after 9 h. Three independent experimental replications were conducted. Values are the means ± SE of three independent experiments (*P* < 0.05). **(B)** Expression of *VvBAP1* in the sensitive grapevine cultivars ‘Chardonnay’ and the resistant varieties ‘Zuoyouhong’ with 40°C treatment. Three independent experimental replications were conducted. Values are the means ± SE of three independent experiments (*P* < 0.05). Lower-case letters above bars denote significant differences attested by Tukey’s HSD test.

In order to evaluate the potential role for *VvBAP1* in the grapevine resistance to heat stress, we first detected the distinction between *VvBAP1* expression profiles in the grapevine varieties with different thermotolerance. The transcript level of *VvBAP1* in the leaves of resistant cultivars ‘Zuoyouhong’ and ‘Beta’ were markedly higher compare to the other two sensitive grapevine cultivars ‘Cabernet Sauvignon’ and ‘Chardonnay’ under non-stressed conditions. After heat stress, the *VvBAP1* transcript level in all grapevine cultivars increased significantly, and *VvBAP1* from ‘Zuoyouhong’ and ‘Beta’ were more sensitive to high temperature, as its transcript level increased much higher compare to that from ‘Cabernet Sauvignon’ and ‘Chardonnay’ (**Figure 1A**). In the follow-up experiment, we further used the tissue culture seedlings of the sensitive cultivars ‘Chardonnay’ and the resistant cultivar ‘Zuoyouhong’ to analyze the expression pattern of *VvBAP1* with 40°C treatment. The results indicated *VvBAP1* was significantly induced in the two grapevine cultivars by high-temperature, showing the highest transcriptional expression at 9 h. Besides, the transcript level of *VvBAP1* in ‘Zuoyouhong’ was always significantly higher than that in ‘Chardonnay’ (**Figure 1B**). These data suggest that *VvBAP1* could play an essential function in heat stress response in the grapevine.

### Effect Of *VvBAP1* Overexpression on the Growth of Transgenic *Arabidopsis* Plants Under Heat Stress

**Figure 2:**
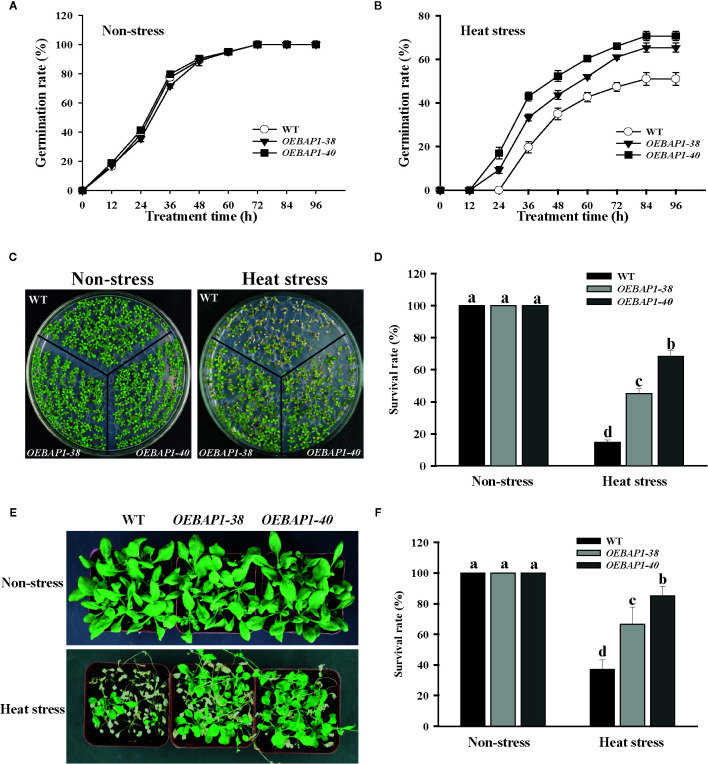
Effects of high temperature on the growth of VvBAP1-overexpressing Arabidopsis plants. Time course of seed germination rates for the wild type and three independent transgenic *Arabidopsis* plants under a normal temperature **(A)** and after heat stress **(B)**. Each experiment was conducted at least thrice and more than 300 seeds were measured in each replicate. Values are the means ± SE of three independent experiments (*P* < 0.05). The phenotype **(C)** and survival rates **(D)** of the wild type and three independent transgenic *Arabidopsis* seedlings under heat stress (45°C for 6 h). The phenotype **(E)** and survival rates **(F)** of the adult wild type and three independent transgenic *Arabidopsis* seedlings under heat stress (42°C for 8 h). Each experiment was conducted thrice. Values are the means ± SE of three independent experiments (*P* < 0.05). Lower-case letters above bars denote significant differences attested by Tukey’s HSD test.

In order to further analyze the physiological function of *VvBAP1* in the heat stress tolerance, the *VvBAP1*-overexpressing *Arabidopsis* plants, which have been reported in our previous research (Hou et al., 2018), were used for seed germination and phenotypic analysis under heat stress. The results of the seed germination rate showed that there was no substantial difference between the seeds of each genotype under non-stressed conditions (**Figure 2A**), when germinated after heat stress treatment, all plants displayed a significant reduction in seed germination percentages, and the two ectopic overexpressing lines showed faster germination rate compared to the wild type (**Figure 2B**). When grown under non-stressed conditions, all the plants exhibited similar phenotypes. However, after heat treatment, the *VvBAP1*-overexpressing *Arabidopsis* exhibited better growth than wild type plants (**Figures 2C, E**), and the transgenic lines displayed obviously higher survival rates compared to the wild type (**Figures 2D, F**). These results indicated that *VvBAP1* did indeed improve resistance to heat stress in plants.

### Effects of *VvBAP1* Overexpression on Physiological Indexes of Transgenic *Arabidopsis* Plants Under Heat Stress

**Figure 3:**
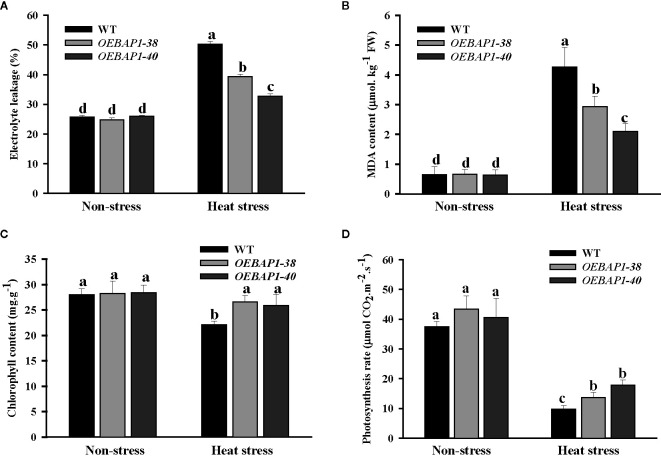
Analysis of physiological indexes of *VvBAP1*-overexpressing *Arabidopsis* under heat stress. The electrolyte leakage **(A)**, MDA content **(B)**, chlorophyll content **(C)**, photosynthetic rate **(D)** in the leaves of overexpressing lines under heat stress (45°C for 2 h). Each experiment was conducted thrice. Values are the means ± SE of three independent experiments (*P* < 0.05). Lower-case letters above bars denote significant differences attested by Tukey’s HSD test.

To further investigate the function of *VvBAP1* in regulating thermoresistance, the electrolyte leakage and MDA content in leaves of *VvBAP1*-overexpressing *Arabidopsis* and the wild type was analyzed. We found that there was no apparent differences in the electrolyte leakage and MDA content between each genotype plants under non-stressed conditions. However, all the plants exhibited a remarkable increase in the MDA content and electrolyte leakage following heat treatment, and this was more significant in wild type, relative to the transgenic plants (**Figures 3A, B**). These findings demonstrated that *VvBAP1* has a positive role in improving the cytomembrane stability to enhance heat resistance in plants further.

Studies have shown the plant photosynthesis can be suppressed early following exposure to high temperature (Larkindale et al., 2005; Allakhverdiev et al., 2008). Given this, we explored the impact of heat stress on chlorophyll content and photosynthetic rate. As shown in **Figure 3C**, under non-stressed conditions, transgenic lines exhibited marginally higher chlorophyll content, relative to the wild type plants. Heat stress exerted no substantial impact on the transgenic plants’ chlorophyll content, but dramatically reduced that of the wild type. The photosynthetic rate of each genotype of plants declined after heat stress treatment. However, the photosynthetic rate of *VvBAP1*-overexpressing lines was considerably higher compared to the wild type (**Figure 3D**). These provide further evidence that the photosynthetic ability of plants overexpressing *VvBAP1* was less affected when compared with the wild type under high-temperature condition, which could be a reason for the *VvBAP1*- mediated increase tolerance to heat stress.

### Changes in the Levels of Accumulated ROS in *VvBAP1*-Overexpressing *Arabidopsis* Leaves Under Heat Stress.

**Figure 4:**
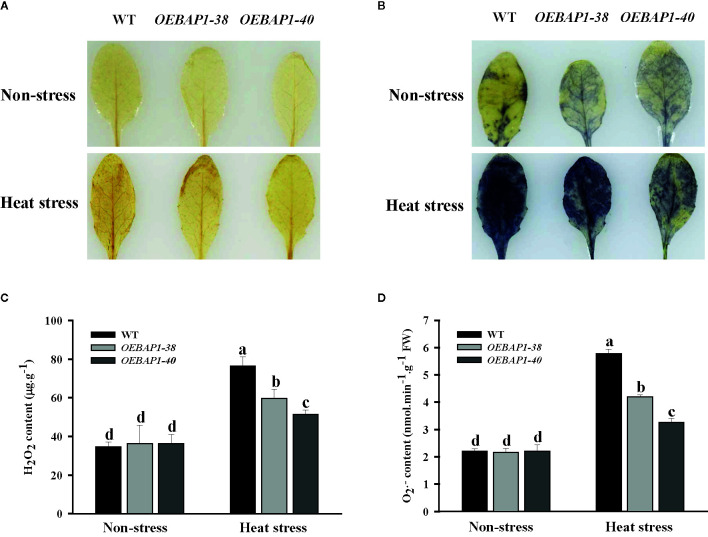
Changes in the levels of accumulated ROS in *VvBAP1*-overexpressing *Arabidopsis* leaves under heat stress. In situ accumulation of H_2_O_2_ **(A)** and $\left({\text{O}}_{2}^{-}\right)$ **(B)** in leaves treated with and without heat stress (45°C for 2 h) revealed by DAB and NBT staining, respectively. Quantitative measurement of H_2_O_2_ **(C)** and $\left({\text{O}}_{2}^{-}\right)$ **(D)** concentrations in leaves treated with and without high temperature with and without heat stress (45°C for 2 h). Each experiment was conducted thrice. Values are the means ± SE of three independent experiments (*P* < 0.05). Lower-case letters above bars denote significant differences attested by Tukey’s HSD test.

When plants are subjected to heat stress, oxidative damage caused by excessive of ROS production was identified as a critical limiting factor in plant growth by disrupting macromolecules and cytomembrane (Miller et al., 2007; Larkindale and Vierling, 2008). Consequently, the effects of heat stress on the contents of H_2_O_2_ and $\left({\text{O}}_{2}^{-}\right)$ were measured by the histochemical detection in the leaves of each genotype plants. When stained separately with DAB and NBT, which were applied to evaluate H_2_O_2_ and $\left({\text{O}}_{2}^{-}\right)$ accumulation, the leaves were similarly and lightly stained under non-stressed condition. In contrast, the leaves of the wild type displayed more intense brown coloration or blue patches, relative to the leaves of transgenic plants after heat stress (**Figures 4A, B**). These results showed that high temperature led to increased ROS production such as H_2_O_2_ and $\left({\text{O}}_{2}^{-}\right)$, while those in transgenic plants were significantly lower, relative to the wild type. These findings were confirmed further by conducting quantitative assays (**Figures 4C, D**).

### Overexpression of *VvBAP1* Enhanced the Activities of ROS-Scavenging Under Heat Stress

**Figure 5:**
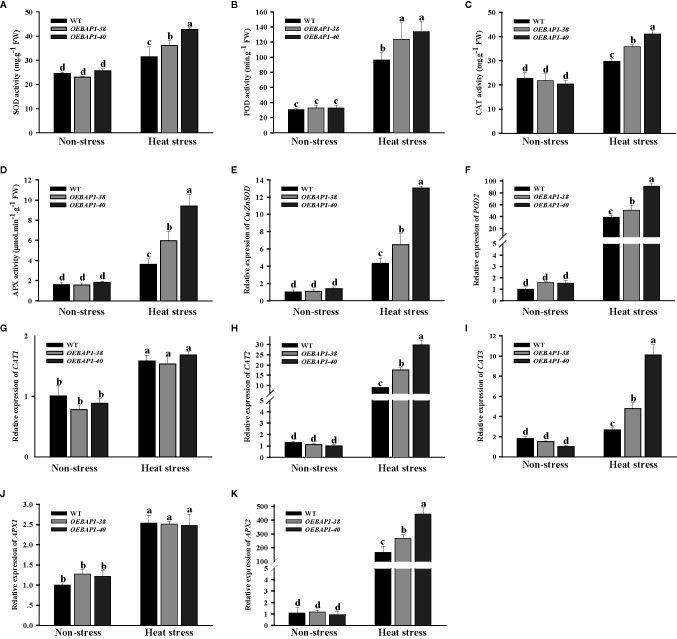
Effects of heat stress on ROS-scavenging enzymes of *VvBAP1*-overexpressing *Arabidopsis* leaves. The activity of CAT **(A)**, POD **(B)**, CAT **(C)** and APX **(D)**, the expression of *SOD1* **(E)**, *POD2* **(F)**, *CAT1* **(G)**, *CAT2* **(H)**, *CAT3* **(I)**, *APX1* **(J)** and *APX2* **(K)** in the leaves of overexpressing lines under heat stress (45°C for 2 h). Each experiment was conducted thrice. Values are the means ± SE of three independent experiments (*P* < 0.05). Lower-case letters above bars denote significant differences attested by Tukey’s HSD test.

As is well known, the measurement of the activity of ROS-scavenging enzymes has been extensively applied to analyze the resistance to stress in plants (Suzuki et al., 2011).The main antioxidant enzymes include SOD, POD, CAT and APX could work together to reduce cellular ROS accumulation and attenuate oxidative injury to plants (Xu et al., 2016). Thus, we assessed the activity of these four enzymes, and the relative expression of their corresponding genes *Cu/Zn SOD*, *POD2*, *CAT1*, *CAT2*, *CAT3*, *APX1* and *APX2*. The results showed that heat stress increased antioxidant activities and up-regulate transcription of their corresponding genes in all plants. Compared with the wild type, overexpression of *VvBAP1* enhanced the increase in antioxidant activities well as their corresponding genes transcription levels induced by heat stress (**Figures 5A–K**). Collectively, these data suggested that overexpress *VvBAP1* enhanced the antioxidant enzyme activity by promoting the expression of genes encoding those enzymes *in vivo*, resulting in reduced levels of ROS under heat stress

### *VvBAP1* Is Involved in Heat Tolerance by Enhancing Heat Response-Related Gene Expression

**Figure 6:**
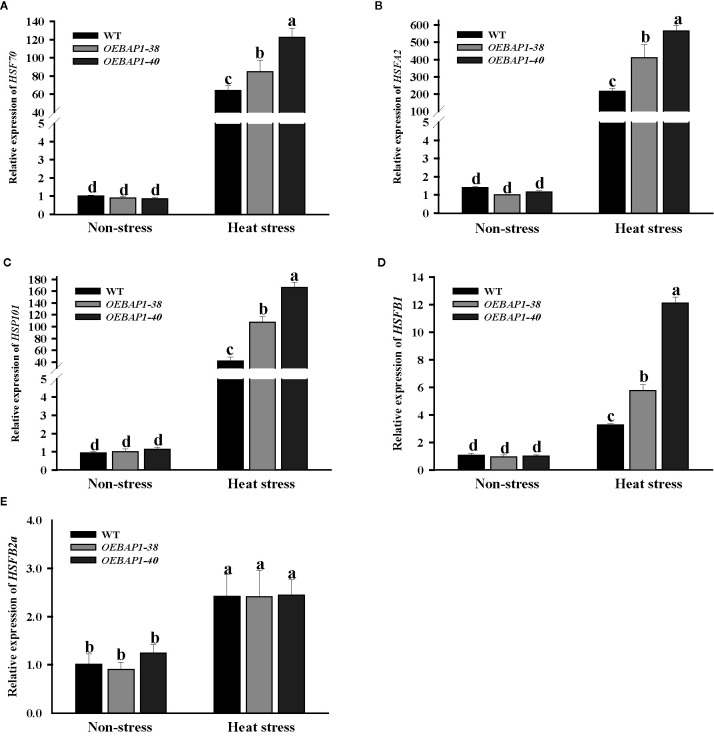
Effects of heat stress on the expression of heat-related genes in *VvBAP1-*overexpressing *Arabidopsis* leaves. The expression of *HSP70* **(A)**, *HSP101* **(B)**, *HSAFA2* **(C)**, *HSAFB1* **(D*)*** and *HSFB2a* **(E)** in the leaves of overexpressing lines under heat stress (45°C for 2 h). Each experiment was conducted thrice. Values are the means ± SE of three independent experiments *(P* < 0.05). Lower-case letters above bars denote significant differences attested by Tukey’s HSD test.

Apart from the antioxidant system, HSPs, often regarded as important molecular chaperones that ensure proper protein folding, which is vital in the growth and development of plants under the high-temperature conditions (Hahn et al., 2012). Moreover, HSFs are related to the direct regulation the transcriptional level of heat stress-induced genes (Baniwal et al., 2004). We assessed the changes in the transcript abundances of *HSP70*, *HSP101*, *HSFA2*, *HSFB1*, and *HSFB2a*. After heat treatment, these genes were markedly up-regulate in all genotype plants, especially *HSP70*, *HSP101*, and *HSFA2*. Their transcript level was induced hundreds of times. Additionally, the transcript abundances of *HSP70*, *HSP101*, *HSFA2*, and *HSFB1* in transgenic plants were much higher compared to the wild type (**Figures 6A–E**). These results suggested that the increased *HSPs* and *HSFs* transcription might be relevant to *VvBAP1*-mediated heat stress response in grapevine, which may have improved the transgenic plants thermoresistance.

## Discussion

Temperature is considered as one of the critical factors directly influencing grapevine during the three primary growth and development stages: sprout, flowering, and fruit-coloring (Lorenz et al., 1995), which are strongly correlated to the yield and quality of grape (Bonnefoy et al., 2013; Bonada and Sadras, 2015; Fraga et al., 2016). However, the grapevine is frequently exposed to a high-temperature condition detrimental to growth (Pereira et al., 2014). Thus, revealing the mechanism of the grapevine thermoresistance is a vital research topic that provides a reliable theoretical basis for grapevine breeding. Increasing data on transcriptomic and proteomic analyses have provided neoteric insights for elucidating the potential molecular mechanism of the grapevine thermoresistance (Liu et al., 2012; Carbonell-Bejerano et al., 2013; Liu et al., 2014; George et al., 2015). However, understanding their precise functions and molecular mechanism in heat resistance of those genes and protein remains clarified at the present time. In this study, the underlying physiological and molecular mechanisms of *VvBAP1* from grapevine were preliminarily explored in response to heat stress.

BAP1 has been demonstrated to belong to C2 domain phospholipid-binding protein; it could suppress programmed cell death in *Arabidopsis* induced by virulent bacterial or oomycete pathogens (Yang et al., 2006; Yang et al., 2007). Later study has shown that low temperatures could induce the *AtBAP1* transcription level elevate in *Arabidopsis* (Zhu et al., 2011). To reveal the biological functions of *VvBAP1* in grapevines, We cloned *VvBAP1* from the resistant grapevine cultivar ‘F-242’and functionally characterized it as the nearest orthologous to *AtBAP1* in our previous reports (Zhang et al., 2014). Then, we found that *VvBAP1* was correlated with the cold response process in grapevines, *VvBAP1* could regulate the soluble sugar content and enhance antioxidant enzyme activities, thereby promoting the grape cold resistance (Hou et al., 2018). In the research presented here, we showed that the elevated expression of *VvBAP1* was higher in the resistant cultivars compare to that from sensitive cultivars under high temperature conditions (**Figure 1A**). Furthermore, The qRT-PCR results indicated *VvBAP1* was significantly induced by high-temperature; the highest level of expression was shown at 9 h (**Figure 1B**). These data imply that *VvBAP1* probably positively affects the grapevine heat stress response. In the follow-up experiment, we further used the *VvBAP1*-overexpressing *Arabidopsis* plants to analyze the physiological functions of VvBAP1 in heat stress response. Seed germination and phenotypic analysis results indicated that overexpression of *VvBAP1* improved *Arabidopsis* thermoresistance (**Figures 2A–F**).

As is well known, the measurement of electrolyte leakage contents in leaves has been extensively applied to analysis plant cell membrane damage under abiotic stresses (Moore and Roberts, 1998). MDA, the end lipid peroxidation product induced by ROS, are widely used as a marker of ROS-mediated injuries for plants (Bajji et al., 2002). The contents of chlorophyll have been commonly utilized to index the heat stress impact on photosynthesis in plants (Zhou et al., 2014; Wang X. L. et al., 2017). Thus, we measured electrolyte leakage, MDA content, chlorophyll contents, and net photosynthetic rate to evaluate the role of *VvBAP1* in the transgenic *Arabidopsis* plants under heat stress. The results found that the wild type showing obviously higher in the cell electrolyte leakage and MDA content compared to the transgenic plants, indicating the positive effects of *VvBAP1* especially on heat resistance in plants by improving the stability of the cytomembrane (**Figures 3A, B**). Additionally, the decreases of chlorophyll concentration and photosynthetic rate in transgenic plants leaves were not as apparent as in the wild type leaves under heat stress (**Figures 3C, D**).

Oxidative damage caused by excessive ROS production was identified as one of the principal limiting factors in plant growth under high temperatures (Hossain, 2015). Previous research has found that increased ROS levels in heat-sensitive rice are significantly more evident compared to the resistant rice under the high-temperature conditions, indicating that ROS accumulation is closely related to thermoresistance in plants (Zhao et al., 2018). In this study, high temperature led to increased ROS production such as H_2_O_2_ and $\left({\text{O}}_{2}^{-}\right)$, while those in transgenic plants markedly decreased, relative to the wild type (**Figures 4A–D**). These data suggested that overexpressing *VvBAP1* suppressed ROS excessive accumulation, which contributed to mitigating oxidative injury to plants caused by heat stress. SOD, POD, CAT, and APX have been recognized as important ROS scavengers to play a crucial function in heat stress response, their activity levels are directly associated with the acquisition of the thermotolerance in plants (Haider et al., 2017). For instance, the mutants that lacked the capacity to eliminate ROS were significantly weaker in basal thermotolerance (Larkindale et al., 2005). In addition, overexpression of *TaFBA1* enhanced-transgenic tobacco basal thermotolerance by improving antioxidant enzyme activity and reducing accumulation of ROS (Li et al., 2018). Similarly, the antioxidant enzyme-encoding genes have been extensively applied to analyze ROS responsive and oxidative stress. In this study, we also found that compared with the wild type, overexpression of *VvBAP1* enhanced the increase in antioxidant activities well as their corresponding gene transcription levels induced by heat stress (**Figures 5A–K**). The results suggested that overexpression of *VvBAP1* increased the antioxidant enzyme activity by promoting the expression of genes encoding those enzymes *in vivo*, leading to reduced ROS level, resulting in enhanced plant thermoresistance.

HSPs, often regarded as critical molecular chaperones that ensure proper protein folding, are essential to help plants acquire thermotolerance under the high-temperature conditions (Hahn et al., 2012). For example, *HSP70* is significantly induced by high temperatures in grapevines (Morrell et al., 1997; Zhang et al., 2005). Besides, HSFs are related to the direct regulation the transcriptional level of heat stress-induced genes (Baniwal et al., 2004). It was previously reported that *VvHsfA1a*, *VvHsfA2a*, *VvHsfB1*, and *VvHsfB2A* were markedly up-regulated in *Vitis vinifera* L during heat stress (Xin et al., 2013; Rocheta et al., 2014). Therefore, we detected the *HSP70*, *HSP101*, *HSFA2*, *HSFB1* and *HSFB2a* expression in transgenic plants. The results of qRT-PCR revealed that the transcript abundances of *HSP70*, *HSP101*, *HSFA2*, and *HSFB1* in *VvBAP1* overexpressing *Arabidopsis* plants were markedly up-regulated compare to the wild type (**Figures 6A–E**). These data suggested that the increased *HSPs* and *HSFs* transcription might be relevant to *VvBAP1*-mediated heat stress response in grapevine.

In summary, we propose that *VvBAP1* may play a potentially important role in enhanced grapevine thermoresistance, mainly by a combination of increased antioxidant enzyme activity and promoted heat stress response genes expression. Heat stress has been shown to induce Ca^2+^ accumulation, in order to regulate *HSPs* transcription and plant thermotolerance (Zhang et al., 2009; Liu et al., 2010). Whether Ca^2+^ regulates the affinity of VvBAP1 binding to its phospholipids substrate and therefore is involved in the heat stress response signaling network, will be necessary to investigate in follow-up experiments. In our previous study, the *VvBAP1* promoter has the MBS element binding with the MYB transcription factor (Hou et al., 2018). The MYB family is one of most crucial transcription factor families involved in regulating the physiological process such as development, metabolism, and stress response in plants (Dubos et al., 2010). There is a need to conduct further studies to determine whether MYB transcription factors involved in response to heat stress in grapevine. If so, can MYB proteins act as regulators and directly bind to the MBS element within *VvBAP1* promoter to enhance *VvBAP1* expression? These research questions will form the subject of future studies.

## Data Availability Statement

The raw data supporting the conclusions of this article will be made available by the authors, without undue reservation.

## Author Contributions

QY performed experiments, interpreted data, and wrote the article. JY and ZZ performed experiments and interpreted the data. XL conceived and designed experiments and edited the article. LH analyzed the data. All authors contributed to the article and approved the submitted version.

## Funding

This research was funded by “National Key Research and Development Program of China (Grant No. 2018YFD1000302)” and “Natural Science Foundation of China (Grant No. 31572107 and 31872082)”.

## Conflict of Interest

The authors declare that the research was conducted in the absence of any commercial or financial relationships that could be construed as a potential conflict of interest.
