# Supplementary material for: VvBAP1, a Grape C2 Domain Protein, Plays a Positive Regulatory Role Under Heat Stress
Source: Front Plant Sci. 2020 Nov 9;11:544374. doi: 10.3389/fpls.2020.544374 (PMC7680865; doi:10.3389/fpls.2020.544374)
Supplement: Supplementary file 1 [file Table_1.docx]

**Supplementary Material**

**Table 1 Real-time PCR primers**

| Gene name | Primer sequence(5’ to 3’) |
| --- | --- |
| *VvBAP1* | FP: CAGGTTGAGGGACGACAAG  RP: CAAACTTGAACCGTCCGATA |
| *VvACTIN* | FP: ATAGAAGCAGCAAGGGA  RP: TGAGGCTCTTACTAATG |
| *AtCu/ZnSOD* | FP: CTGCATCTCTACTGGACCTC  RP: CCACATCCAACTCTCGAGC |
| *AtPOD2* | FP: GTTTACCCGACCCTACACTC  RP: ATCCTATTCATTGCCTCCAC |
| *AtCAT1* | FP: GTCCTGGGATTCAGACAGGC  RP: GGCCTCACGTTAAGACGAGT |
| *AtCAT2* | FP: ATCCTTACAAGTATCGTCCAGCTAG  RP: CTGAGACCAGTAAGAGATCCAGATA |
| *AtCAT3* | FP: AGGCCAATCTCCATATAAGC  RP: ACGACCACCATAAATCTTAG |
| *AtAPX1* | FP: GCTGGAGGGGTCGCATT  RP: CAAATCGCAACCCACCG |
| *AtAPX2* | FP: GATAGGTTCTCCATTCTCTTTAGG  RP: TCTTGTACTCTTCTTTCACTTCCG |
| *AtHSFA2* | FP: CGCCTTTCCATTGGGACA  RP: GTAGCATTACCATAACTTAGACCGC |
| *AtHSFB1* | FP: AAGACTGTTCAAACCAGCGTGT  RP: CCTTCTGGACCTTCTTCACCTTT |
| *AtHSFB2a* | FP: TGTTCTTTTTCAGTTTTGTTTTCTTC  RP: TCCCTACCTCATTTCTCGTTTATTA |
| *AtHSP70* | FP: TTTGCTTGAGATTCTAGTTGGTTTC  RP: GTCTTTCATAGGTCAGAGCGAGTT |
| *AtHSP101* | FP: AACTCAACGAAACAAACCCAG  RP: ATTGTCTCGTTTGTCTTGTGTGT |
| *AtACTIN* | FP: GGTAACATTGTGCTCAGTGGTGG  RP: CACGACCTTAATCTTCATGCTGC |
